# Case Report: Multiple Strokes and Digital Ischemia in a Young COVID-19 Patient

**DOI:** 10.4269/ajtmh.20-1101

**Published:** 2020-11-17

**Authors:** Harsh Shah, Aditya Iyer, Raja Zaghlol, Sandeep Raparla

**Affiliations:** Department of Internal Medicine, Medstar Washington Hospital Center, Georgetown University, Washington, District of Columbia

## Abstract

COVID-19 is an infectious disease caused by SARS-CoV-2. This enveloped RNA coronavirus primarily has tropism for the respiratory tract. However, it has also been shown to have various extrapulmonary manifestations such as pulmonary embolism, ischemic strokes, deep venous thrombosis, or arterial thrombosis. We present a case of a 34-year-old woman who had severe COVID-19 infection with no respiratory symptoms and developed strokes in multiple vascular territories and digital ischemia due to thrombosis formation in the brachial circulation of her arm despite receiving therapeutic anticoagulation.

## INTRODUCTION

The first case of SARS-CoV-2 was reported in China. Common symptoms include fever, cough, muscle pain, and fatigue, and nearly 80% of patients have normal or decreased white blood cell counts, with many presenting with lymphocytopenia.^1^ One of the complications of this virus is its induction of hypercoagulability which is not completely understood. From the Virchow’s triad, it can be suspected that a hypercoagulable state results from fluctuation or manipulation of circulating prothrombotic factors such as Lupus anticoagulant (LA) and fibrinogen. Endothelial injury by the virus has also been reported as a trigger for the hypercoagulable state. Our patient had severe COVID-19 infection with strokes in multiple vascular territories and suffered from digital ischemia due to clot formation in brachial circulation of her arm.

## CASE REVIEW

**Figure 1.**
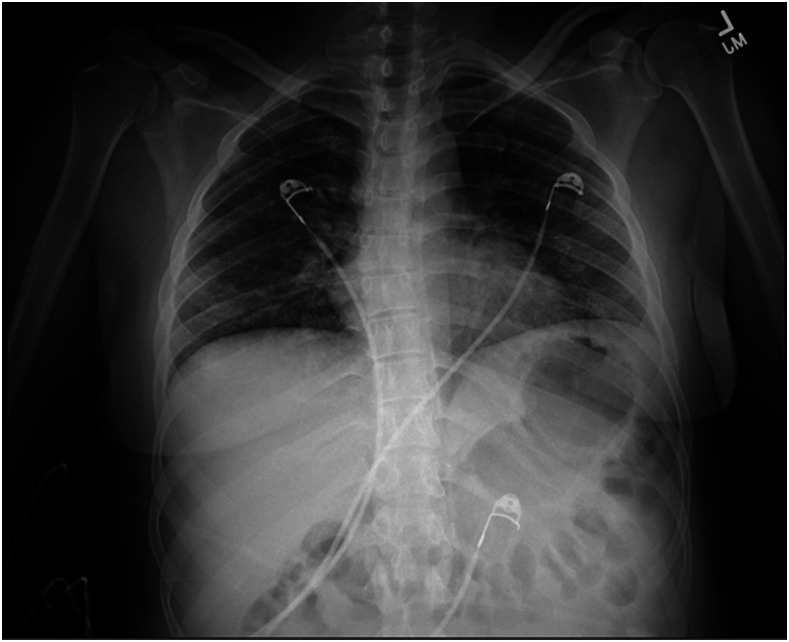
A portable chest X-ray shows a normal cardiac silhouette. There is no focal consolidation or effusion. Costophrenic angles are present. Trachea is midline.

**Figure 2.**
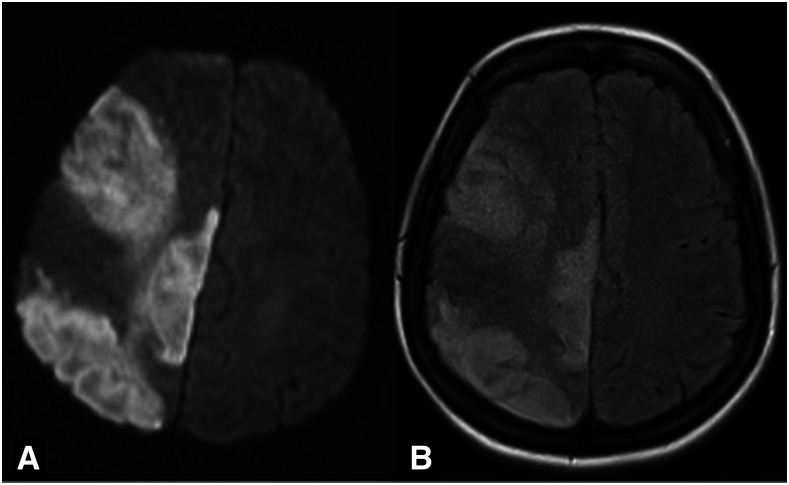
Magnetic resonance imaging (MRI). (**A**) shows confluent diffusion-weighted imaging hyperintensities of the right frontal temporal lobe, right temporal parietal, and frontal lobe with panel (**B**) MRI showing corresponding fluid-attenuated inversion recovery hyperintensities.

We present a case of a 34-year-old Hispanic woman who presented with fatigue, loss of appetite, and malaise in addition to headache and tingling sensation in her fingers. At the time of presentation, she did not have any complaints regarding subjective fevers at home, shortness of breath, chest pain, cough, loss of smell, abdominal pain, nausea, vomiting, or any changes in bowel habits. Her past medical history was notable for insulin-dependent diabetes mellitus type I and hyperlipidemia, with no history of atrial fibrillation or other prothrombotic diseases. Her home medications included both short- and long-acting insulin along with atorvastatin 80 mg. She was not on any oral contraceptive medications. Initial laboratory values and vital signs in the emergency department were pertinent for a heart rate of 121 beats/minute, blood oxygen saturation of 98% on room air, glucose of 515 mg/dL, anion gap of 13, presence of beta-hydroxybutyrate, C-reactive protein of 17.5 mg/L, fibrinogen of 886 mg/dL (213–536 mg/dL), D-dimer of 2.54 mcg/mL (0–0.5 mcg/mL), and ferritin of 170.6 ng/mL (5–148 ng/mL).^1–3^ A portable chest X-ray (CXR) on admission revealed a normal cardiac silhouette with no signs of focal consolidation or effusion (Figure 1). She was found to have diabetic ketoacidosis, and workup for possible triggers revealed an infectious etiology. Her COVID-19 reverse transcriptase–PCR test (RT-PCR) nasal swab specimen resulted positive. Her CXR did not show pneumonia, and urinalysis was negative for infection. She did not receive any treatment for coronavirus infection such as remdesivir, hydroxychloroquine, glucocorticoids, or plasma because of lack of hypoxemia and symptoms including cough, dyspnea, loss of smell, and other viral symptoms. She was started on intravenous insulin infusion and placed on low molecular weight heparin (LMWH) for deep venous thrombosis prophylaxis regimen during the first few hours of admission. Her mental status continued to decline with decorticate posturing of the left upper extremity. Magnetic resonance imaging of the brain showed acute ischemic strokes in the right middle cerebral artery (MCA) and anterior cerebral artery (ACA) vascular territory with the development of cerebral edema (Figure 2). She did not receive tissue plasminogen activator because of being outside the treatment window. However, the patient’s mental status continued to worsen with medical management, warranting emergent surgical right frontotemporal decompressive hemicraniectomy.

**Figure 3.**
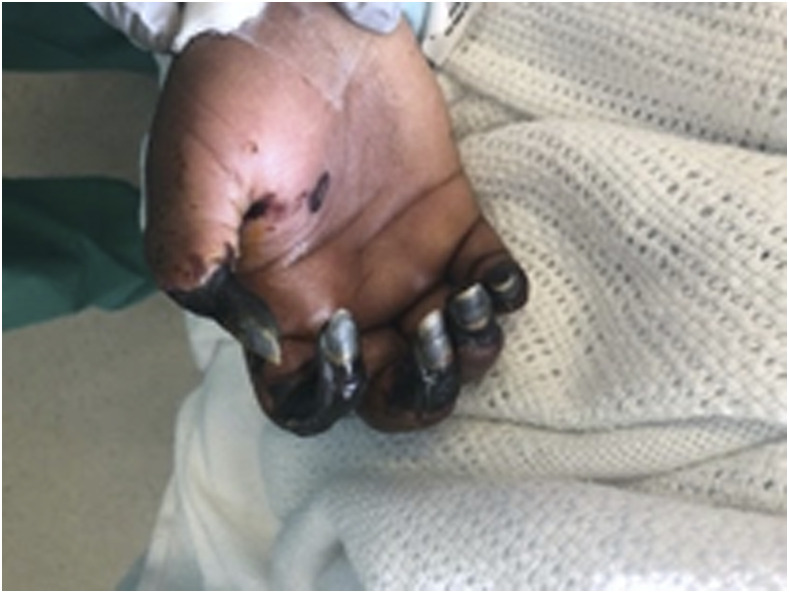
Photograph of the volar hand exhibiting dry gangrenous digits from digital ischemia. This figure appears in color at www.ajtmh.org.

Multiple strokes in a young female prompted further hypercoagulable workup which revealed positive LA screen with elevated dilute Russell viper venom time (DRVVT) at 63.9 seconds (36.1–50.8 seconds), DRVVT screen/confirm ratio 1.31 (0.97–1.22) with normal levels of beta-2 glycoprotein I Ab IgM, IgG, and IgA. Multiple days after surgery, her course was complicated by the slow development of right-hand swelling, for which the arterial duplex showed radial artery occlusion and monophasic flow in the ulnar artery. Computed tomographic angiography of the right upper extremity showed poor to non-opacification of the radial, ulnar, and palmar arch vessels and irregularities of the brachial arteries, suggesting mural thrombus. Despite therapeutic anticoagulation with unfractionated heparin (UH), her right-hand swelling worsened to dry gangrene of right digits (Figure 3). Initially, there was no acute surgical intervention performed for dry gangrene and eventually was managed with therapeutic dosing of LMWH. She was scheduled for eventual amputation of the ischemic fingers but later suffered a pulseless electrical activity arrest (PEA) and was not able to be resuscitated despite cardiopulmonary resuscitation. We suspect the leading differential for her PEA is pulmonary embolism, given her hypercoagulable state.

## DISCUSSION

The most salient feature of this case includes the predominant symptom burden of SARS-CoV-2 infection manifesting as thrombosis in an otherwise healthy young woman with no past medical history of coagulopathy. As per Oxley et al.,^2^ which reported large-vessel stroke as a presenting feature in the young population, three of the five cases mentioned the involvement of the MCA as a major vascular territory. This is further supported by the data published in the global COVID-19 stroke registry where it reported MCA as the most frequently affected vascular territory.^3^ In addition to the ischemic stroke presentation, the top presenting complaints were dyspnea/hypoxia.^4^ Our patient’s stroke involved both the MCA and ACA. Another salient feature is despite having tropism for the lungs, she had no respiratory symptoms as evidenced by imaging and clinical presentation.

As per Merkler et al.^4^ which compared risk of ischemic stroke in patients with COVID-19 versus patients with influenza, it mentions the association of the virus with vigorous inflammatory response accompanied by coagulopathy, with elevated *D*-dimer levels and frequent presence of antiphospholipid antibodies. Our patient also had elevated *D*-dimer levels and LA antibody screenings, indicating a prothrombotic state. We acknowledge that these antibodies can transiently rise in any critical illness, but multiple case reports now have documented that in addition to elevated *D*-dimer levels, antiphospholipid antibodies have also been elevated in COVID-19 patients.^5^ However, given the ultimate demise of the patient, repeat serologic confirmation of LA laboratory markers at 12 weeks could not be retested to confirm or refute an underlying antiphospholipid syndrome.

Several autopsy studies showed that COVID-19 affects arterial and venous blood vessels of various sizes and capillary beds but does not symmetrically involve all tissues.^6^ One of the theories that appear to have been playing a role is the selectivity of coronavirus toward angiotensin-converting enzyme 2 (ACE2) receptors.^7^ On binding to cell surface ACE2 receptors, it inhibits the vaso-protective functions of ACE, causing pro-inflammatory and prothrombotic states. In addition, the systemic increase of pro-inflammatory cytokines such as interleukin-6 is also believed to be a major contributor of inducing hypercoagulable state in COVID-19. Furthermore, COVID-19 compromises the integrity of endothelial monolayer by causing endothelial cell death through its lytic replication,^7^ thus exposing the thrombogenic basement membrane and in turn leading to the activation of coagulation cascade.^7^

Acknowledging that SARS-CoV-2 leads to a hypercoagulable state, the role of anticoagulation is important. As per Klok et al.,^8^ the incidence of thrombotic complications in critically ill patients in intensive care unit is 31%. The American Society of Hematology recommends LMWH as venous thromboembolism prophylaxis agent over UH. Literature has varied in terms of dosing, selection, and timing of therapeutic agents. Some recommend using therapeutic LMWH if there is a rise of the inflammatory markers and *D*-dimer on days 7–14.^9^ Others recommend the use of scoring calculators such as sepsis-induced coagulopathy published by the International Society of Thrombosis and Hemostasis, which uses platelet count, international normalized ratio, and sequential organ failure assessment scores to risk-stratify and guide anticoagulation strategies.^10^ Our patient received both prophylactic and therapeutic doses of anticoagulation during the hospital course and developed digital ischemia despite receiving therapeutic anticoagulation.

In conclusion, we report a COVID-19 patient who did not develop pneumonia and had no history of hypercoagulable condition developing multiple arterial thrombosis involving the neurovascular and peripheral vascular system with both leading to grave sequelae, despite receiving anticoagulation. Our case reveals that more research needs to be undertaken to understand the cascade of events leading to a prothrombotic state and the role of anticoagulation regimen for prevention and treatment of these highly susceptible thrombotic events.
